# Outcomes of Pregnancies in Liver Transplant Recipients: Experience of a Single Center in Turkey

**DOI:** 10.34172/aim.2022.128

**Published:** 2022-12-01

**Authors:** Firat Tulek, Alper Kahraman, Kamil Yalçın Polat

**Affiliations:** ^1^Department of Midwifery, Faculty of Health Sciences, Uskudar University, Istanbul, Turkey; ^2^Department of Obstetrics and Gynecology, Memorial Atasehir Hospital, Istanbul, Turkey; ^3^Department of Obstetrics and Gynecology, Haseki Training and Research Hospital, Istanbul, Turkey; ^4^Department of General Surgery and Organ Transplantation Center, Memorial Atasehir Hospital, Istanbul, Turkey

**Keywords:** Liver transplantation, Pregnancy, Perinatal outcome, Rejection

## Abstract

**Background:**

Liver transplantation is the ultimate treatment for end-stage liver failure. As organ donation systems improve, more reproductive-age women are expected to undergo liver transplantation. Current studies indicate increased risk of some perinatal and maternal complications; however, the available data is still scarce. Therefore, we aimed to evaluate the maternal and fetal outcomes of pregnancies in liver transplant recipients.

**Methods:**

We retrospectively evaluated liver transplantations performed between 2011 and 2020 in a tertiary center. Perinatal, maternal, fetal outcomes and transplant status were assessed among pregnancies conceived after liver transplantation.

**Results:**

Among 1137 patients, 82 (7.2%) were reproductive-age females. Ten pregnancies in nine patients were identified after liver transplantation. The mean age of patients was 29.3±6.1 at transplantation, and 32.5±5.4 at conception. The mean interval between conception and transplantation was 30.3±11.7 months. There were eight live births (80%), one miscarriage (10%) and one termination (10%). Three patients delivered<37^th^ gestational week (37.5%). The median gestational age at birth was 38.5 (IQR: 5.21) weeks. The mean birth weight of infants was 2669.3±831 g. Two patients were diagnosed with preeclampsia (25%) and acute graft rejection episode was observed in one patient (10%) during pregnancy.

**Conclusion:**

Although the incidence of some perinatal complications, such as hypertensive disorders and preterm delivery, is increased in liver transplant recipients, pregnancy after liver transplantation appears to have favorable outcomes for the mother, fetus and transplant with close monitoring by a multidisciplinary team.

## Introduction

 Liver transplantation is a life-saving procedure and the ultimate treatment for end-stage liver failure. The number of successful liver transplantations has greatly increased in the last few decades and developments involving immunosuppression, surgical techniques and organ preservation have increased the life expectancy of recipients.^1^ Around 30% of liver transplant recipients are female, one-third of whom are of reproductive age and 15% of whom are pediatric recipients with 70% expected to survive through reproductive ages.^2^ Most women with end-stage liver disease suffer from amenorrhea and anovulation due to impairments in the hypothalamic-pituitary-gonadal axis.^3^ However, the effects of advanced liver disease on reproductive functions and sexuality subside within a few months after transplantation that renders reproductive-age female liver transplant recipients able to conceive.^4^ Therefore, nowadays pregnancies in liver transplant recipients are not uncommon. Studies about these pregnancies generally indicate favorable outcomes.^5^ Nevertheless, some complications such as increased risk of acute cellular rejection, graft loss, preeclampsia, preterm delivery and intrauterine growth restriction have been identified in addition to increased anxiety in recipients regarding the well-being of the child and their own health status.^5-7^ Hence, these are considered as high-risk pregnancies that require monitoring in a tertiary center by a multidisciplinary team and successful outcomes strictly depend on close observation and careful management.^5,6^ Several studies have provided information about the maternal and fetal outcomes of these pregnancies; however, the accumulated data is still limited. In this study, we evaluated the maternal and perinatal outcomes of 10 pregnancies in 9 liver transplant recipients.

## Materials and Methods

 We retrospectively reviewed the data from patients who underwent liver transplantation between 2011 and 2020 in a single tertiary transplantation center of Memorial Atasehir Hospital affiliated with Uskudar University in Istanbul/Turkey. Among these patients, women who conceived after liver transplantation and received perinatal care or delivered were included in the study. The demographic characteristics of patients including transplantation indication, donor type, age at transplantation, immunosuppression, and concomitant diseases of recipients were obtained from hospital records.

 The maternal data and obstetric history of recipients were obtained either from patient records or via phone contacts with each patient when required. Pregnancy outcomes such as perinatal and obstetric complications, interval between pregnancy and transplantation, mode of deliveries, gestational ages at delivery and delivery indications were evaluated.

###  Statistical Analyses 

 Statistical analyses were carried out using IBM SPSS version 23. Shapiro-Wilk test and quantile-quantile (Q-Q) plot were used to determine the distribution of data. Mean ± standard deviations were calculated for normally distributed data. Median and interquartile ranges (IQR) were calculated for data with skewed distribution.

## Results

**Figure 1:**
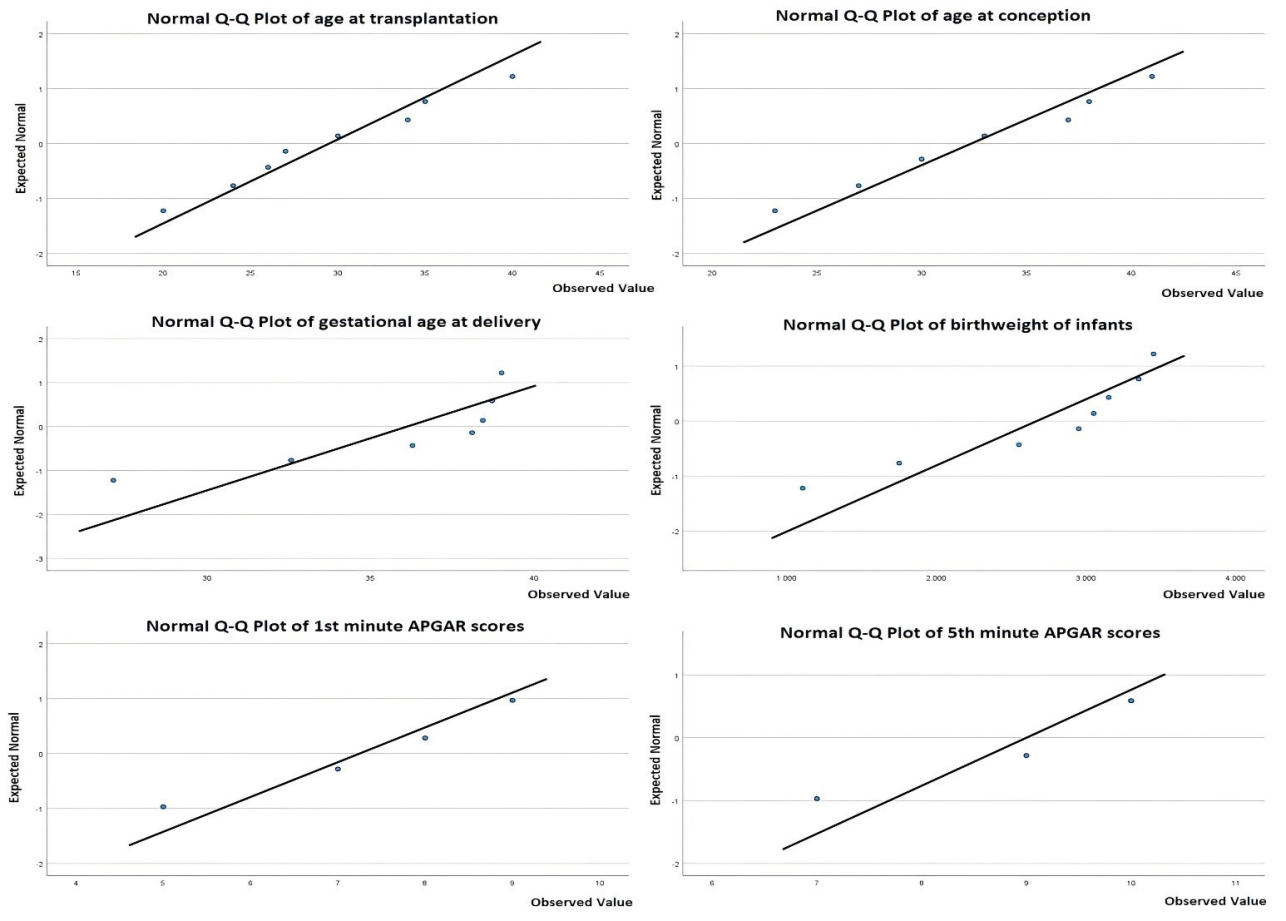
Normal Q-Q Plot Graphic of Distribution of Variables

 Overall, 1137 patients were found to undergo liver transplantation in our institution within the selected period of time. One hundred forty-five (24.9%) liver transplant recipients were between 15 and 45 years of age, 82 (7.2%) of whom were female. One hundred thirty-nine (12.2%) patients were under 15 years of age, 59 (5.1%) of whom were female. A total of nine liver transplant recipients were found to have conceived and 10 pregnancies were identified. The distribution of variables of these patients is given in Figure 1.

 Eight of the patients received live donor liver transplantation, one patient received cadaver donor transplantation. The mean age at transplantation was 29.3 ± 6.1 years. Transplantation indications were: chronic hepatitis B infection (44.4%), Wilson’s disease (22.2%), Budd-Chiari syndrome (11.1%), metastatic neuroendocrine tumor (11.1%) and cryptogenic cirrhosis (11.1%). None of the patients had a history of graft rejection before their pregnancies. All of the patients were on immunosuppressive tacrolimus therapy and were also receiving ursodeoxycholic acid (UDCA) before, during and after the pregnancies. One patient (Patient 6) was receiving tenofovir for chronic hepatitis B infection and two patients were taking levothyroxine for concomitant hypothyroidism. One patient (Patient 2) was diagnosed with premature ovarian failure 2 years before transplantation and one patient (Patient 4) had homozygote Factor V Leiden mutation. The demographic characteristics of patients/pregnancies are given in Table 1.

**Table 1:** Demographic Characteristics of Patients/pregnancies

**Patient/Pregnancy**	**Transplantation Indication**	**Donor Type**	**Age at Transplantation (y)**	**Age at Conception (y)**	**Transplantation-Pregnancy Interval (mon)**	**İmmunosuppressive Therapy/Other Medications**	**Obstetric history **	**Co-morbidity**
1	Wilson’s disease	Live	20	23	34	Tacrolimus /UDCA	G1P1001	None
2	Wilson’s disease	Live	28	31	29	Tacrolimus /UDCA	G0P0000	Premature ovarian failure
3	Metastatic neuroendocrine tumor	Live	27	30	42	Tacrolimus /UDCA	G0P0000	None
4	Budd Chiari Syndrome	Live	26	30	40	Tacrolimus /UDCA	G2P1101	Homoztgote Factor V leiden mutation
5a	HBV	Live	35	35	8	Tacrolimus /UDCA, thyroxin	G0P0000	Hypothyroidism
5b	HBV	Live	35	38	46	Tacrolimus /UDCA, thyroxin	G1P0010	Hypothyroidism
6	HBV	Live	40	41	16	Tacrolimus /UDCA, tenofovir	G7P5025	None
7	HBV	Cadaver	30	33	35	Tacrolimus /UDCA	G0P0000	None
8	Cryptogenic	Live	24	27	28	Tacrolimus /UDCA	G0P0000	None
9	HBV	live	34	37	25	Tacrolimus /UDCA	G1P1001	None

HBV, hepatitis B virus; UDCA, ursodeoxycholic acid.

 The mean age at conception was 32.5 ± 5.4 years and the mean interval between transplantation and conception was 30.3 ± 11.7 months. Nine out of 10 pregnancies were conceived spontaneously, one pregnancy was conceived by *in vitro* fertilization/oocyte donation. Eight of these 10 pregnancies resulted in live births whereas one pregnancy that was conceived by *in vitro* fertilization/oocyte donation (Patient 2) resulted in miscarriage and one pregnancy (Patient 5/a) was terminated in the first trimester for maternal health concerns due to the short interval between liver transplantation and conception. Pregnancy outcomes, maternal fetal and perinatal complications are summarized in Table 2.

**Table 2:** Pregnancy Outcomes, Maternal, Fetal and Perinatal Complications in Liver Transplant Recipients

**Patient/Pregnancy**	**Mode of Conception**	**Gestational Age at Delivery (wk/d)**	**Pregnancy Outcome **	**Mode of Delivery/Indication**	**Birthweight (g)**	**Fetal Gender**	**Apgar Scores**	**Ante Natal Complications**	**Post-partum Complications**	**Fetal Complications/Malformations**	**Graft Rejection After Pregnancy**	**Status of the recipient (Years After Delivery)**
1	Spontaneous	38 wk 5 d	Live birth	CS/maternal request	3050	F	8/10	None	None	None	None	Alive (1)
2	IVF/oocyte donation	8 wk 3 d	Miscarriage	n/a	n/a	n/a	n/a	n/a	n/a	n/a	None	Alive (1)
3	Spontaneous	38 wk 3 d	Live birth	CS/maternal request	3350	M	9/10	Urinary tract infection (*Klebsiella*)	None	None	None	Alive (1)
4	Spontaneous	27 wk d	Live birth	CS/abruptio placentae, history of uterine rupture	1105	M	5/7	Acute rejection episode, abruptio placentae	Surgical site infection (MRSA)	Prematurity, Neonatal intensive care unit admission 75 days	None	Alive (2)
5a	Spontaneous	7 wk 6 d	Termination	n/a	n/a	n/a	n/a	n/a	n/a	n/a	None	Alive (6)
5b	Spontaneous	39 wk 0 d	Live birth	CS/ maternal request	3450	F	8/10	None	None	None	None	Alive (3)
6	Spontaneous	38 wk 5d	Live birth	CS/ maternal request	2950	F	9/10	None	None	None	None	Alive (5)
7	Spontaneous	38 wk 5 d	Live birth	CS/ maternal request	3150	F	7/9	None	None	None	None	Alive (1)
8	Spontaneous	32 wk 4 d	Live birth	CS/severe preeclampsia	1750	F	5/7	Preeclampsia	None	Prematurity, NICU 5 days	None	Alive (2)
9	Spontaneous	36 wk 2 d	Live birth	CS/severe preeclampsia	2550	F	7/9	Preeclampsia	None	None	None	Alive (3)

CS, cesarean section; F, female; M, male; n/a, not applicable; NISU, Neonatal intensive care unit.

 The mean birth weight of infants was 2669.3 ± 831 g. There were two (25%) low birth weight infants in deliveries; however, all infant birth weights were between the 10^th^ and 90^th^ percentile regarding their gestational ages at delivery. The median first minute APGAR score of infants was 7.5 (IQR: 3.25) and the median fifth minute APGAR score was 9.5 (IQR: 2.50). All infants were delivered via cesarean section. Two women (25%) underwent cesarean section due to severe preeclampsia, one patient due to placental abruption (12.5%) and the remaining five were on maternal request (62.5%). The median gestational age at birth was 38.5 (IQR: 5.21) weeks in our study population.

 One patient (Patient 4) presented with generalized pruritis and four-fold elevated transaminase levels in the 12^th^ gestational week of her pregnancy. Her anamnesis revealed that she had omitted taking tacrolimus throughout that week. The presenting condition was interpreted as acute graft rejection episode. Following re-initiation of immunosuppressant therapy, her symptoms disappeared and liver transaminase levels reduced to normal values. This patient (Patient 4) delivered in the 27^th^ week of pregnancy owing to placental abruption. Patient 4 was revealed to have bad obstetric history prior to transplantation. She was found to be G2P0201 before transplantation. Her first pregnancy resulted in stillbirth due to placental abruption and her second pregnancy was delivered via emergent laparotomy due to spontaneous uterine rupture in the 34^th^ gestational week. She also reported a family history of placental abruption in her sister’s pregnancies.

 Two pregnancies were complicated by hypertensive disorders (Patients 8, 9) and both of these pregnancies delivered < 37^th^ gestational week by cesarean section due to severe preeclampsia.

 One pregnancy (Patient 3) was diagnosed with uncomplicated urinary tract infection in the first trimester. *Klebsiella pneumoniae* was isolated in urine culture and treated with fosfomycin.

 None of the patients received blood transfusion in the postpartum period. Two infants were admitted to the neonatal intensive care unit due to prematurity. No structural malformations were observed in any infants and no patients showed any sign of graft rejection following their deliveries until the commencement of this study.

## Discussion

 Currently, evidence with satisfactory rigor is lacking primarily due to scarcity of data to propose precise management guidelines for pregnancies in liver transplant recipients. In this study, we demonstrated that outcomes of pregnancies in liver transplant recipients are favorable regarding mother, fetus and allograft under careful close monitoring by a multidisciplinary team.

 Clear recommendations about timing of conception following liver transplantation are yet to be determined. Currently, all of the authors are in agreement that conception should be delayed at last 1 year after liver transplantation.^2,6,8-10^ Pregnancies conceived within 1 year after liver transplantation have significantly higher rates of adverse maternal and fetal outcomes as well as four-fold increase in graft rejection risk.^11^ Live birth rate was found as low as 41% in pregnancies conceived within 1 year after liver transplantation.^12^ Some studies indicate increased incidences of very low birth weight infants, higher graft rejection in pregnancy and within 2 years after delivery in patients conceived between 1–2 years following transplantation.^13^ Therefore, some authors recommend to delay pregnancy 2 years after transplantation as the safest option.^14^ In our study population, there was one pregnancy conceived within one year after transplantation (Patient 5a). All of the patients presented in our study were counseled about timing of a possible pregnancy after liver transplantation and offered contraception. This pregnancy (Patient 5a) occurred due to barrier contraception failure and terminated in the first trimester regarding increased graft rejection risk. The remaining patients conceived at least 1 year after transplantation.

 A study conducted by Westbrook et al showed 73% live birth, 10% termination and 23% miscarriage rates in liver transplant recipients’ pregnancies.^11^ Similarly, Deshpande et al showed 76.9% live birth and 15.6% miscarriage rates among pregnancies in liver recipient women.^2^ Interestingly, live birth rates were higher and miscarriage rates were lower in liver transplant recipients compared to the general population in both Europe and North America.^2^ Parallel to these findings, 8 out of 10 pregnancies (80%) resulted in live birth, one pregnancy (10%) resulted in miscarriage and one pregnancy (10%) resulted in termination in our study population.

 Preeclampsia is defined as a new-onset hypertension after the 20^th^ gestational week concomitant with proteinuria or new sign of end organ failure. Development of preeclampsia and hypertension risks are increased in liver transplant recipients, which are respectively shown to complicate 19% and 37% of pregnancies after liver transplantation.^13^ Hypertensive complications in pregnancies after liver transplantations are attributed to the vasoconstrictive effects of immunosuppressant agents and possible underlying renal dysfunction.^15^ Among immunosuppressant agents, tacrolimus is found to be associated with a smaller increase in hypertensive complication risk in comparison to steroids or cyclosporine.^16^ Westbrook et al demonstrated a significant association between prematurity, low birth weight and preeclampsia in liver transplant recipients, indicating medically indicated iatrogenic preterm delivery of infants.^11^ All of the patients were under tacrolimus monotherapy in our study. Although none of our patients developed pregnancy-induced hypertension, 2 out of 10 pregnancies (20%) were complicated with severe preeclampsia, both of whom delivered < the 37^th^ week of pregnancy for maternal safety.

 Calcineurin inhibitors are known to be associated with glucose intolerance, and the incidence of gestational diabetes mellitus (GDM) in liver transplant recipients ranges between 0% and 11% in several studies.^6,17^ A population-based study conducted by Ghazali et al found significantly higher GDM rates in liver transplant recipients (8.6%) in comparison to the general population (5.4%).^18^ Cautious monitoring of GDM in pregnancies after liver transplantation is emphasized in some previous studies.^9^ All of the patients presented in this study were screened for insulin resistance by 75 oral glucose tolerance test in the 24^th^ gestational week and none of them were found to have GDM. Absence of GDM occurrence in our cases could be a result of our small sample size. On the other hand, all of our patients were receiving UDCA before, during and after the pregnancy. This is a consequence of the routine practice of UDCA administration to liver recipients following transplantation as an institutional adoption. UDCA is a steroid bile acid with known anti-oxidative and anti-inflammatory properties and used extensively in cholestatic liver diseases.^19^ Furthermore, some recent studies indicate probable anti-diabetogenic effects of this agent on glucose homeostasis and insulin resistance.^20-23^ The anti-diabetogenic properties of UDCA might have contributed to lack of GDM cases among our patients. However, studies with larger populations are required to clarify this issue.

 In a previous survey, Kubo et al demonstrated that pregnancy outcomes in liver recipients are not affected by donor type.^24^ Most of the liver transplants in Western countries such as Spain, the United Kingdom and United States are obtained from deceased donors.^25^ In contrast, in Asian countries, liver transplantations are usually performed with transplants obtained from living donors.^25^ Consistent with these findings, in our study, 9 out of 10 pregnancies were reported in living donor transplant recipients whereas only one pregnancy (Patient 7) was related to a cadaveric donor.

 Pregnancy does not seem to negatively affect liver graft functions. Acute graft rejection in liver transplant recipients’ pregnancies is between 10% and 17% and loss of graft as a result of acute rejection episode in pregnancy is uncommon.^8,17^ However, alterations of serum immunosuppressant levels could be expected in pregnancy. Acute graft rejection episodes in pregnancy are mainly attributed to non-compliance with immunosuppressant therapy or hemodilution due to increased plasma volume and subsequent reduction of serum immunosuppressant levels.^17^ For instance, serum tacrolimus levels may increase in pregnancy due to hepatic cytochrome P450 enzyme inhibition whereas cyclosporine levels may decrease owing to increased hepatic clearance.^6^ Regarding these factors, serum immunosuppressant levels are recommended to be monitored frequently throughout pregnancy, once every 4 weeks until the 32^nd^ gestational week of pregnancy, bi-weekly between the 32^nd^ and 36^th^ weeks of pregnancy and weekly from the 36^th^ week till delivery.^10,26^ Parallel with previous studies, we observed one case (10%) of acute graft rejection episode among our patients, caused by maternal non-compliance with immunosuppressant therapy who recovered by re-initiation of tacrolimus in the first trimester of pregnancy.

 Preterm delivery defines the pregnancies delivered before the 37^th^ gestational week. Previous studies show that the incidence of preterm deliveries in liver transplant recipients are increased compared to the normal population, ranging from 14% to 53%.^2,11,13,17,26^ In a recent meta-analysis, Prodromidou et al reported the rate of preterm delivery as 32% in liver recipients.^14^ This increase was ascribed to higher incidence of prenatal complication in these patients like preeclampsia.^27^ In our study population, three patients were delivered before the 37^th^ gestational week. Consistent with the results of previous studies, the preterm delivery rate was found at 37.5%. Two of these pregnancies were delivered due to severe preeclampsia and one of them for placental abruption.

 Tacrolimus is a calcineurin inhibitor, widely used in solid organ transplant recipients for immunosuppression and classified as “Category C” by U.S. Food and Drug administration.^26^ Although tacrolimus increases the incidences of some complications such as hypertensive disorders in pregnancy, maternal diabetes, renal dysfunction and perinatal fetal hyperkalemia, the incidence of fetal malformation (4.4%) in tacrolimus receiving patients was not significantly different from the general population (3-5%) and it is generally considered safe in pregnancy.^8^ All of our patients were receiving tacrolimus monotherapy for immunosuppression throughout pregnancy and none of the infants were shown to have any type of congenital defects among the study population.

 A study conducted by Coffin et al showed an approximately two-fold increase in postpartum hemorrhage and blood transfusions in liver recipients, regardless of the mode of delivery.^27^ Conflicting with their findings, none of our patients required blood transfusions in the postpartum period. Studies indicate higher rates of genitourinary infections during pregnancy and postpartum wound complications in liver transplant recipients in comparison to normal pregnancies, probably as a consequence of immunosuppressive therapy.^18^ In our study, there was one case of postpartum wound infection (12.5%) among deliveries and one case of urinary tract infection (10%) in the course of pregnancy.

 In our study, all of the infants were delivered via cesarean section. Five of these eight cesareans were performed on maternal request (62.5%). Cesarean rates in liver transplant recipients are shown to be two to three folds higher in comparison to the general population.^27,28^ Furthermore, Coffin et al demonstrated that cesarean rates have been following an increasing trend in liver transplant recipients over the last 20 years, which is probably a cumulative effect of maternal attitudes and changing obstetric concerns based on medico-legal liabilities.^27^ On the other hand, Yoshimura et al pointed out the high levels of maternal anxiety in pregnancies of liver transplant recipients and emphasized the importance of proper counselling.^7^ In the absence of any obstetric contraindication, vaginal delivery in liver transplant recipients is not associated with unfavorable maternal or fetal outcomes and it is the preferred mode of delivery.^8,29,30^ It is hard to determine the exact rates of cesarean delivery on maternal request; however, studies throughout the world indicate an incidence of about 2.5% to 3%.^31,32^ We found extraordinarily high cesarean rates in our study population and most of these cesareans were performed due to maternal concerns (62.5%), highly exceeding the reported rates in the general population. Proper maternal counselling and reassurance of liver recipients about safety of vaginal delivery could reduce cesarean rates in these patients.

 In conclusion, pregnancy outcomes in liver transplant recipients appear to be favorable; however, patients should be monitored cautiously for immunosuppressant levels, hypertensive disorders and preterm deliveries with a multidisciplinary approach. Additionally, they should be counseled properly in terms of timing of delivery, contraception and anxiety.
